# Mobile Mental Wellness Training for Stress Management: Feasibility and Design Implications Based on a One-Month Field Study

**DOI:** 10.2196/mhealth.2596

**Published:** 2013-07-10

**Authors:** Aino Ahtinen, Elina Mattila, Pasi Välkkynen, Kirsikka Kaipainen, Toni Vanhala, Miikka Ermes, Essi Sairanen, Tero Myllymäki, Raimo Lappalainen

**Affiliations:** ^1^VTT Technical Research Centre of FinlandTampereFinland; ^2^Department of PsychologyUniversity of JyväskyläJyväskyläFinland

**Keywords:** stress, mental health, mobile phone, acceptance and commitment therapy, field studies, user experience, design

## Abstract

**Background:**

Prevention and management of work-related stress and related mental problems is a great challenge. Mobile applications are a promising way to integrate prevention strategies into the everyday lives of citizens.

**Objective:**

The objectives of this study was to study the usage, acceptance, and usefulness of a mobile mental wellness training application among working-age individuals, and to derive preliminary design implications for mobile apps for stress management.

**Methods:**

Oiva, a mobile app based on acceptance and commitment therapy (ACT), was designed to support active learning of skills related to mental wellness through brief ACT-based exercises in the daily life. A one-month field study with 15 working-age participants was organized to study the usage, acceptance, and usefulness of Oiva. The usage of Oiva was studied based on the usage log files of the application. Changes in wellness were measured by three validated questionnaires on stress, satisfaction with life (SWLS), and psychological flexibility (AAQ-II) at the beginning and at end of the study and by user experience questionnaires after one week’s and one month’s use. In-depth user experience interviews were conducted after one month’s use to study the acceptance and user experiences of Oiva.

**Results:**

Oiva was used actively throughout the study. The average number of usage sessions was 16.8 (SD 2.4) and the total usage time per participant was 3 hours 12 minutes (SD 99 minutes). Significant pre-post improvements were obtained in stress ratings (mean 3.1 SD 0.2 vs mean 2.5 SD 0.1, *P*=.003) and satisfaction with life scores (mean 23.1 SD 1.3 vs mean 25.9 SD 0.8, *P*=.02), but not in psychological flexibility. Oiva was perceived easy to use, acceptable, and useful by the participants. A randomized controlled trial is ongoing to evaluate the effectiveness of Oiva on working-age individuals with stress problems.

**Conclusions:**

A feasibility study of Oiva mobile mental wellness training app showed good acceptability, usefulness, and engagement among the working-age participants, and provided increased understanding on the essential features of mobile apps for stress management. Five design implications were derived based on the qualitative findings: (1) provide exercises for everyday life, (2) find proper place and time for challenging content, (3) focus on self-improvement and learning instead of external rewards, (4) guide gently but do not restrict choice, and (5) provide an easy and flexible tool for self-reflection.

## Introduction

### Background

Work-related mental health problems are the most common cause of disability in the countries of the Organization for Economic Cooperation and Development [1]. Stress is strongly associated with mental health problems, such as depression [2]. In 2005, 22% of European workers reported suffering from stress [3], and stress is estimated to be a significant factor in 50-60% of lost working days [4].

Preventative interventions targeted at individuals at risk may be used to contain the costs and suffering related to stress and mental disorders [5,6]. Occupational stress management programs usually focus on teaching individuals techniques to cope with stress, and are delivered in sessions over several weeks [7,8]. Especially interventions based on cognitive-behavioral techniques have been found effective, but as they are usually delivered in group sessions by trained professionals, they are also relatively costly [5,8].

Technology-assisted prevention programs enable easier, earlier, and more flexible access to care at a lower cost, and many of them have been shown to reach equal effectiveness to face-to-face therapies in the treatment of psychological problems [9]. In the past few years, due to the wide availability of smartphones and mobile apps, mobile delivery of interventions has become feasible. Mobile phones facilitate integrating interventions into the daily lives of the citizens, allow unobtrusive monitoring of their activities and contexts, and make it possible to provide interventions at opportune moments, that is, when most needed and desired [10].

### Acceptance and Commitment Therapy in Stress Management

Most computerized therapies build on psychological therapy methods, usually cognitive-behavioral therapies (CBT) [9]. The so-called third wave of CBT includes mindfulness and acceptance based behavioral and cognitive therapies, such as acceptance and commitment therapy (ACT). ACT aims to increase the individual’s psychological flexibility, which is “the ability to contact the present moment more fully as a conscious human being, and to change or persist in behavior when doing so serves valued ends” [11].

Psychological flexibility can be increased through the six core processes of ACT [11,12]. *Acceptance* means embracing feelings and events without trying to change them. *Cognitive defusion* techniques try to change the way one relates to thoughts, for example, by observing one’s thoughts and feelings without being caught up in them. The skill of *being present* helps to experience psychological and environmental events in a non-judgmental way and bringing full awareness to the present moment. The *self-as-context* process aims at becoming aware of the flow of personal experiences with an understanding that they are not one’s essence. *Values* determine what is truly important and help choose life directions. *Committed actions* are concrete actions chosen based on personal values. [11,12] To train the skills, an eclectic mix of metaphor, paradox, and mindfulness methods, along with a wide range of experiential exercises and value-guided behavioral interventions are used [11,13].

Psychological flexibility is associated with better mental health and behavioral changes [11,12]. ACT-based interventions have been found effective in reducing work stress and increasing work performance and the ability to innovate [14-17].

### Mobile Apps for Mental Wellness

Mobile interventions have been used effectively to promote physical health, but despite increasing activity in the area, mobile mental wellness apps are still in their infancy [18]. Most apps developed thus far are not guided full-length programs, but rather implement a range of intervention techniques, including mood assessments, experience sampling, and small exercises. The trials have mostly been carried out with small samples, making the efficacy of different interventions and generalizability of the results difficult to assess. Still, previous studies yield several useful insights to inform the design of mental wellness interventions that utilize mobile technology in preventative or therapeutic context.

CBT-based interventions commonly use monitoring of symptoms and mood states as a strategy to develop self-awareness and coping abilities [19]. Mood charting with a mobile and online symptom tracking tool, Mobile Mood Diary, has been explored in a series of small studies carried out with teenagers with mental health problems. The findings suggested that mood charting with mobile phones could increase adherence to therapy and improve self-awareness. In addition, coupling mood charts with a diary could help to support recall and to broach difficult topics with a therapist [19].

Several mobile interventions, such as Mood Map and PRISM, have combined monitoring of mental states with brief exercises [20,21]. Mood Map is a mobile app for increasing emotional self-awareness [20]. The app consists of mood-related experience sampling and CBT-inspired psychological exercises that could be completed in a minute or less. The app has been evaluated in a one-month study among 8 working-age adults who reported moderate or high level of stress [20]. The participants increased their emotional awareness and started practicing new strategies to cope with stress, although some of the impact may be due to the weekly interviews conducted during the study rather than the app. The findings indicated that exercises were more likely to be used when experiencing intense emotions.

In therapeutic context, PRISM is a mobile intervention that was evaluated in a two-week study among 10 outpatients with bipolar disorder [21]. The intervention, delivered via a personal digital assistant and based on Life Goals psychoeducation, prompted users to engage in personally selected self-management behaviors as a response to specific self-reported mood states. The trial resulted in a decrease in depressive symptoms and high satisfaction with the intervention. Several improvements to support long-term use were also brought up, including the ability to enter text entries and offering a broad collection of self-management strategies.

Some interventions have combined mobile and Web apps that serve different functions. Mobilyze! is a mobile phone and Web-based intervention for depression [22]. It uses phone sensors and experience sampling to gather data on users’ contexts and moods and to remind them to use the website. The intervention consists of behavioral skills training through 9 weekly 15-minute lessons on an interactive website, and telephone coaching to increase adherence. The system was trialed with 8 participants who had a diagnosis of major depressive disorder [22]. Their depressive symptoms decreased over the course of the intervention and they were generally satisfied with the program. Despite several technical difficulties such as battery drainage and inaccuracy of sensor data, context-aware interventions seem potentially promising in helping a person gain self-awareness and encouraging behavioral changes at right moments.

One of the few larger studies is the trial of myCompass, a mobile phone and Web-based program for stress, depression, and anxiety [18]. The program consists of self-tracking, reminders, and tips on the phone and CBT-based 10-minute self-management modules on the website. Participants of the 6-week trial were 44 adults experiencing a current episode of depression, anxiety, or stress. The results showed a reduction in psychological distress, and improvements in functional impairment and self-efficacy. The participants appreciated the accessibility and convenience of the app, but wished for more personalized instructions.

Randomized controlled studies of mobile mental wellness apps, especially in preventative context, appear to be scarce. One of the few examples is the evaluation of MEMO, a 9-week mobile intervention for preventing teenage depression consisting of mobile messages and a mobile website [23]. The evaluation of the intervention involved 418 teenage participants in the intervention group. Good user acceptance and perceived usefulness were found, but measured outcomes related to depression have not been published yet. A common critique of the intervention was the large amount of messages; the intervention delivered 2 messages per day over 9 weeks, which appeared to be too frequent for most.

To our knowledge, the only ACT-based mobile intervention reported thus far was by Ly et al [24] who presented a program with Web and mobile components, aimed at supporting individuals to live consistently with their values. The Web component includes psychoeducation and exercises for analyzing personal values. The mobile app reminds the user to perform behaviors in line with their values, gives feedback on their progress, and allows them to view other users’ actions. The 4-week trial of the app included 11 participants with no diagnosed mental disorders. Increased psychological flexibility and value-based actions were measured at post-test, but no effects were found for depression, anxiety, or stress. The qualitative findings of the study suggest that the mere presence of the icon of the app on the mobile phone screen may have increased awareness of values and behaviors [24].

Conclusive evidence of effectiveness of existing mobile mental wellness apps is not yet available, but the results have been promising and several large-scale randomized controlled trials are currently underway [18,23,25]. Based on the studies so far, mobile phone interventions appear to be feasible and convenient for the users, and the mobile phone might in itself have a small positive effect on self-awareness. Nevertheless, there is insufficient knowledge available of what works in mobile mental health promotion and what makes an app successful in producing a lasting change in the user’s wellness. Lessons learned from apps used as a part of therapy may not necessarily translate directly to preventive context.

### Guidelines for Mental Wellness Apps

Several guidelines exist for designing technological apps that engage, motivate, and support behavior and attitude changes. Systems “designed to change people’s attitudes or behaviors” are called persuasive technologies [26] and some guidelines for their design have been developed [26-28]. Gamification, that is, using game-like elements has also been proposed in the domain of wellness apps to increase adherence and engagement [29,30]. However, the guidelines that are based on apps promoting physical health and thus they may not be directly applicable to the domain of mental wellness.

The guidelines that are more closely related to mental wellness apps include Morris’ 7 guidelines for behavior change [31]: (1) remind people who they want to be, (2) foster an alliance (empathy, coinvestigation, joint problem solving), (3) apply social influence, (4) show people what they could lose, (5) put the message where the action is, (6) raise emotional awareness, and (7) reframe challenges. In addition, Doherty et al [32] introduced and explored a set of design strategies to reduce attrition in Web-based interventions and concluded that the interventions need to be: (1) interactive, (2) personal, (3) supportive, and (4) social. Furthermore, using naturally calming elements and providing positive feedback on user actions can minimize stressors for users [33]. Finally, technology should be seen as a tool to assist in the change process; engagement with the treatment rather than with the technology should be the overall aim [34].

### Objectives of the Current Study

In this paper, we describe Oiva, a mobile app for stress management, and present results of a one-month field study that was performed to assess the feasibility of Oiva and validate its design choices. To the best of our knowledge, Oiva is the first ACT-based, stand-alone mobile intervention for self-administered preventative training of stress management skills. Most of the previous studies of mobile mental wellness apps have either focused on mood charting or combined mood charting with brief exercises that have been Web-based in some of the studies. Although mood charting appears to increase adherence and improve self-awareness among certain target groups, none of the studies so far have done a detailed analysis of how mental wellness exercises on a mobile platform should be designed to maximize adherence, user satisfaction and outcomes. To shed light specifically on this matter, the current study excluded the mood-charting component and focused on a structured training program with experiential exercises. The detailed objectives of this study were to: (1) investigate how the participants used the app, (2) study the effects of Oiva on the participants’ mental wellness, (3) explore the user experiences of Oiva and its functions, and (4) derive preliminary design implications for mobile mental wellness apps.

## Methods

### Mobile App

Oiva is a mobile mental wellness-training app targeted at working-age people who suffer from stress. Oiva is a stand-alone app for Android mobile phones and tablets, and it was designed in cooperation of experts in psychology, user needs and technology. The content and logic of Oiva were built upon ACT principles and methods, and the app delivers a complete ACT-based intervention program in bite-sized daily sessions.

Oiva contains 4 intervention modules or “paths” named Aware Mind, Wise Mind, Values, and Healthy Body. The first 3 paths teach the user the 6 core processes of ACT and the fourth path focuses on physical wellness, but with an ACT-based approach. The paths consist of altogether 46 text and audio exercises. *Aware Mind* contains exercises on awareness of the present moment, breathing, and observing one’s body, mind, and surroundings. *Wise Mind* teaches skills related to observation and acceptance of one’s thoughts and feelings. *Values* focuses on clarifying one’s personal values and committing to concrete actions to pursue them. *Healthy Body* includes relaxation, mindful eating, and mindful physical activity exercises.

**Figure 1:**
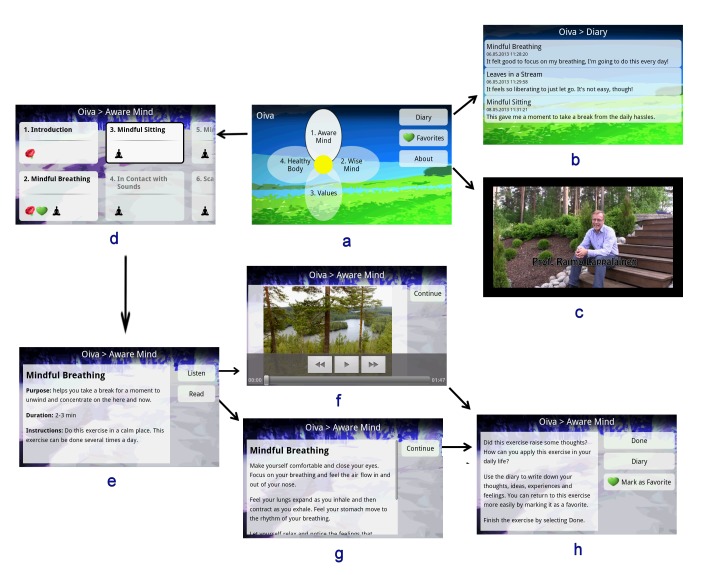
Screenshots of Oiva. (a) the main screen, (b) diary, (c) introduction video, (d) top menu of Aware Mind, (e) exercise introduction screen, (f) audio exercise, (g) text exercise, (h) exercise reflection screen.

Figure 1 presents examples of the user interface of Oiva. The main screen (Figure 1a) of the app contains a flower-shaped menu through which the different paths can be accessed. The main screen also provides an access to the diary (Figure 1b), list of favorite exercises, and an introduction to the app as text and video (Figure 1c). The introduction video informs the users about the purpose of the app and motivates them to use it. The video features an expert in ACT and thereby aim to increase the credibility of the app, as suggested by the persuasive systems design model [27], and creating a feeling of therapeutic alliance, proposed by Morris [31].

Each petal represents one of the paths, which are numbered according to their recommended order. Each path consists of 1–4 subsections (“steps”), which include 5–8 exercises (Figure 1d). An introduction as text and video is included in each path and step, informing the user about the processes and skills taught in them.

Oiva gently steers the user through the intervention program without restricting free navigation. Paths, steps, and exercises are numbered in the recommended order and the next suggested item is dynamically highlighted (Figure 1a and d). However, all paths and exercises are accessible from the very beginning. This design solution follows the tunneling and reduction principles of persuasion [26,27], but in a non-restrictive way.

Most of the exercises are short and take about 1-3 minutes to complete (Figure 1e-h). This aims at making them easy to perform in any situation. Each exercise begins with an introduction presenting the purpose, duration and instructions of the exercise (Figure 1e). The user can choose to do the exercise by listening (Figure 1f) or reading (Figure 1g). After each exercise, a reflection screen (Figure 1h) summarizes the skills learned in the exercise and enables the user to write notes and reflections in the diary (Figure 1b). The notes are saved in the diary and can be accessed later to encourage self-reflection as well as raise emotional awareness, as suggested by Morris [31]. The user can also mark the exercise as a favorite, thus adding it on the list of favorites, which is accessible quickly through the main view.

Progress in the program is presented in several ways. First, the number of completed exercises is displayed for each step. Second, the background color of steps and exercises changes once they are completed. Each completed exercise is rewarded by a virtual rose (Figure 1d), providing immediate graphical feedback of progress [32]. The visual theme of Oiva draws from nature. The graphics and background images depict nature, animals, and landscapes to provide calming elements, as proposed by Moraveji and Soesanto [33]. Pictures, audio, and video are used to make the experience of using Oiva pleasurable and less demanding, reducing the amount of text in a similar manner as done by Ly et al [24].

### Study Participants and Recruitment

Fifteen volunteer participants were recruited via email from the staff of the local technical university in Finland. Thirteen of the participants had not encountered Oiva and did not know the researchers prior to the study. Two of the participants had seen an earlier version of Oiva, because they had volunteered in a short usability test in a laboratory. There were no specific inclusion criteria, only that the participants needed to be interested in stress management and willing to use a prototype mobile app. The app was described as an acceptance-, value-, and mindfulness-based self-help program designed to help in, for example, stress management and relaxation, increasing physical activity, and practicing mindful eating.

The 15 participants (9 female) were university staff, including, for example, a human resources manager, a secretary, a researcher, and a laboratory engineer. Five participants were younger than 30 years old, 5 were between 31 and 40 years, and 5 were older than 40 years.

The participants signed an informed consent form prior to the study and were aware of their right to withdraw from the study at any time. Ethics committee approval was not acquired as the study was deemed to involve minimal risk and the focus was on studying mainly user experiences.

### Procedures

The participants filled in baseline questionnaires and attended a face-to-face group kick-off session, which consisted of two 10-minute presentations, one on ACT theory and one on Oiva. The aim and process of the study were explained. An Android smartphone with Oiva pre-installed was provided for each of the participants to be used for one month. The phone model was either ZTE Blade (7 participants) or Sony Ericsson Xperia ray (8 participants). A short user guide about Oiva was provided on paper. Active, preferably daily use was recommended, but finding personally appropriate ways of use was also encouraged. The study period was one month in May 2012. At the end of the study, each participant was given two movie tickets to compensate for the time spent in study procedures.

### Measures

Data were collected from 3 sources: (1) online questionnaires completed at baseline, after one week’s use and after one month’s use, (2) interviews conducted after one month’s use, and (3) the usage log of Oiva app.

Background information (eg, age and previous experience in using mobile phones and wellness technologies) was collected at baseline. Wellness questionnaires were administered at baseline and after one month’s use. Psychological flexibility was assessed with a 7-item Acceptance and Action Questionnaire (AAQ-II, [35]). AAQ-II employs a 7-point Likert scale from 1 (strongly disagree) to 7 (strongly agree) and includes negative statements, such as “Worries get in the ways of my success”. AAQ-II is scored from 7 to 49, and higher scores suggest less psychological flexibility. Results across seven samples with a total of 3280 participants have provided promising evidence as to the adequate structure, reliability, and validity of the AAQ-II [36]. The Satisfaction with Life Scale (SWLS, [37]) also employs a 7-point Likert scale and includes five positive statements, for example “in most ways my life is close to ideal”. The SWLS is scored from 5 to 35, where higher scores suggest higher satisfaction with life. The SWLS has been found to have good validity and reliability and has been shown to correlate with measures of mental health [37,38]. The single-item stress scale [39] has one statement: “Stress means a situation in which a person feels tense, restless, nervous or anxious or is unable to sleep at night because his/her mind is troubled all the time. Do you feel this kind of stress these days?” The response is recorded on a 5-point Likert scale varying from 1 (not at all) to 5 (very much). The stress scale has shown satisfactory content, criterion, and construct validity for group level analysis [39]. Experienced wellness benefits were measured by user experience questionnaires after one week’s use and one month’s use. Three questions measured Oiva’s perceived usefulness in the maintenance and improvement of wellness, learning new skills, and gaining new insights. These questions were recorded with scales ranging from 1 (completely disagree) to 7 (completely agree).

Qualitative data on user experiences, usage, and usefulness were gathered in individual, semi-structured, face-to-face interviews after one month’s use. The interviews were designed among the authors and a discussion guide outlining the themes of the interview was created. Some of the themes included were: the situations where Oiva was used in, whether using Oiva affected the participant’s wellness, the user experiences of different features of Oiva, Oiva’s ability to persuade and reward usage, and desired new features of Oiva. The first author, who has several years of experience in user experience studies, conducted the interviews. Each interview lasted about 45 minutes and was audio recorded.

The usage log files were collected from the phones after the interview. The usage log contained all user actions and their time stamps. The number of characters in diary entries was also logged, but not the content of the entries.

### Data Analysis

The log files of 14 participants were analyzed. One participant was excluded due to unreliable time stamps in her log file caused by a date change in her phone. Individual usage sessions were first detected and counted. Then, usage days were calculated as days containing a usage session. The durations of individual sessions were calculated based on the start and end times of each session, and the total duration of use by summing the durations of all sessions. As the app can be always “on” and running in the background, there were no specific log events that would flawlessly indicate the start of a usage session. Thus, two criteria were used to determine the start of a session. First, if an “app started” event was detected without any prior activity for the past 10 minutes, a new session was considered to have started. Second, if there was at least a 20-minute pause between consecutive log events, a new session was marked. The 20-minute requirement was chosen to avoid marking a new session while the user was listening to or otherwise performing an exercise.

Statistical analysis of the quantitative data from the wellness questionnaires and our custom rating scales for the experienced benefits was performed as follows. First, one female participant was excluded as she omitted some of the questionnaires. Coincidentally, she was the same participant who was also excluded from log file analyses. Then, change in participants’ ratings of wellness from before to after using Oiva was analyzed with paired comparisons using Wilcoxon signed rank tests. Finally, we tested if the median ratings of experienced benefits of Oiva (ie, improvement or maintenance of wellness, learning new things, and gaining new insights) were statistically significantly on the positive side (ie, above the mid-point of 4) using Wilcoxon signed rank tests.

The interview audio recordings were transcribed and analyzed with a qualitative content analysis method called thematic coding [40]. The data were categorized under three main themes (usage habits, perceived benefits, user experiences of Oiva and its functions). Under each main theme, several subthemes were identified, for example, usage situations, barriers of use, and benefits.

## Results

### Participant Characteristics

According to the baseline questionnaire, all except one of the participants used mobile phones daily, and all but one had used mobile phones for more than ten years. Eleven participants were currently using a smartphone. Of the participants, 67% (10/15) had already tried some mobile wellness apps (eg, for exercise tracking), and 73% (11/15) had tried wellness-related Web services (eg, for weight management). Eleven participants (73%) had some prior knowledge of ACT related topics (eg, mindfulness).

### Usage

The average duration of the usage period from the first log event to the last was 34.0 (SD 5.3, range 26-46) days. The participants used Oiva, on average, on 11.5 (SD 5.8, range 4-20) days, and there were, on average, 16.8 (SD 9.0, range 5-36) usage sessions per participant during the study. The average duration of usage sessions was 12.3 (SD 5.2, range 4.4-24) minutes. The average total usage time per participant was 192 (SD 99, range 56-339) minutes.

### Effects on Wellness

Table 1 presents the participants’ average ratings of stress (score of Elo’s stress scale), life satisfaction (score of SWLS), and psychological flexibility (score of AAQ-II). The change from before to after using Oiva was statistically significant for ratings of stress (*z*=3.00, *P*=.003) and life satisfaction (*z*=2.32, *P*=.02). There was no statistically significant change in psychological flexibility (*z*=0.06, *P*=.950).

**Figure 2:**
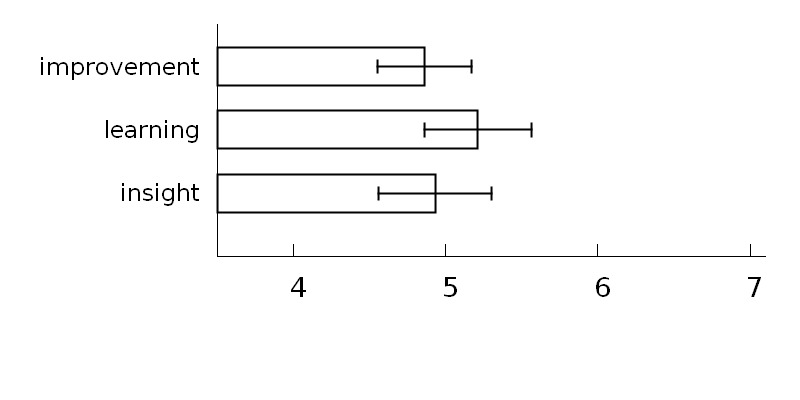
Mean post-study ratings (± SEM) of the effects attributed to Oiva.

Figure 2 presents the mean ratings of benefits that the participants reported having gained from using Oiva. All mean ratings were on the positive side. Observed medians for each scale were 5 and statistically significantly higher than the mid-point of 4 for all the scales, improvement or maintenance of wellness (*z*=2.29, *P*=.022), learning new skills (*z*=2.67, *P*=.008), and gaining new insights (*z*=2.17, *P*=.03).

**Table 1:** Ratings of stress and scores in SWLS and AAQ-II questionnaires before and after using Oiva.

	Before mean (SEM^a^)	After mean (SEM)	*P*
Stress	3.1 (0.2)	2.5 (0.1)	.003
SWLS	23.1 (1.3)	25.9 (0.8)	.02
AAQ-II	17.2 (1.5)	17.2 (1.6)	.95

^a^SEM=standard error of mean

### Usage Habits and Barriers

The most common place to use Oiva, reported by 80% (12/15) of participants, was at home. Typical usage contexts at home included: in bed before falling asleep or after waking up (5/15, 33% of participants), in the backyard (2/15, 13% of participants), or in the middle of household routines (3/15, 20% of participants). One participant who used Oiva outdoors commented that the elements of nature (eg, birds singing or fresh air) strengthened the effects of the exercises. Only 27% (4/15) of participants had used Oiva at work. Forty percent (6/15) of the participants had tried performing exercises in mobile contexts, for example, when commuting, and 27% (4/15) of them reported liking this way of use. Mobile situations were often regarded as restless and not providing a possibility for proper concentration.

Despite little actual mobile use, the mobile device was regarded as a good platform for a stress management app. Ten participants (67%) brought up the ease of carrying a mobile phone along as the main benefit. Even at home, the mobile phone was taken along to different places, such as bed, hammock, or living room floor, where the usage of a computer would have been more difficult. Also, being able to open the app quickly and not requiring an extra device to carry were important benefits of the mobile phone for 27% (4/15) of participants (eg, compared to relaxation CDs or laptops). The mobile phone implementation was perceived especially suitable for short and easy exercises (6/15, 40% of participants) and for audio exercises (4/15, 27% of participants). Three participants specifically mentioned that some of the exercises, such as long exercises requiring concentration and tranquility, were less suitable for a mobile phone. However, not having Oiva installed in their personal phone affected use; 60% (9/15) of participants believed that, if Oiva had been installed in their own phone, they would have used it in a more versatile way and also in mobile contexts.

A typical barrier of use was being busy in everyday life (7/15, 47% of participants), which made it difficult to find suitable, peaceful moments for exercises. For example, concentrating on the exercises in the evening when already tired was perceived challenging (3/15, 20% of participants). Forty percent (6/15) of participants also mentioned that they forgot to use Oiva unless they consciously dedicated a time slot for it in their schedules.

The participants preferred short and simple exercises that were easy to integrate into their everyday life, could be done anywhere and were easy to perform even without Oiva after learning the techniques. Forty-seven percent (7/15) of participants said that these exercises gave them immediate benefits and that they intended to continue doing them also after the study.

The one-month usage period seemed too short to 73% (11/15) of the participants. They felt that establishing profound lifestyle changes and new routines takes longer than a month, and that they only had time to explore the exercises but not to apply the skills properly to their own life. The participants who had learned to apply the skills in their life and perform the exercises even without Oiva had been familiar with the techniques already prior to the study. The short breathing and relaxation exercises were an exception as the techniques were very easy to learn.

### Perceived Benefits

According to the interviews, the main benefit of Oiva was being able to take a break in the midst of daily hassles, whether at work or at home (eg, taking care of children or household chores). Especially the short breathing and relaxation exercises were found useful for this purpose. One participant described how differently her day began after doing an exercise compared to her routine mornings: “When I did an exercise in the morning before leaving for work, the day begun in a totally different way. Otherwise I would have just driven to work in a rush”*.* The participants who had done exercises in bed at night felt that it helped them fall asleep easier.

Four participants (27%) reported gaining more profound benefits, such as changes in their thoughts and attitudes. These included getting a new perspective on their life or starting to consider their values and actions. Two participants (13%) had also discussed their values with their spouses. One of them stated this would not have happened without Oiva. The other one had experienced value-based exercises beneficial in a new life situation, after becoming a parent for the first time. A third participant described that value exercises had helped her realize concretely that she has the power to decide how she spends her time and her life.

Four participants (27%) also reported gaining the ability to let go of their thoughts and feelings, and change their perspective to them. They reported that these skills had helped them deal with negative thoughts and feelings, distance themselves from stressful thoughts and accept setbacks. It had made them question whether some of their issues were as serious as they had thought.

### User Experiences of Oiva and Its Functions

Mindfulness was considered a good philosophy for Oiva. Seven participants (47%) commented that Oiva’s exercises concretely demonstrated how they could apply mindfulness in their everyday life. In general, the participants regarded the exercises as interesting, concrete, down-to-earth, and memorable. The stories and metaphors helped them understand the topics. In addition, as Oiva included versatile topics and exercises in a structured form, the participants were able to choose the most suitable exercises.

Audio exercises were preferred over text by 73% (11/15) of participants because they provided guidance through the exercise. They were also perceived to provide a “more personal feeling” and make it easier to concentrate and relax. An unexpected benefit, reported by one participant, was that audio exercises enabled performing them together with other people. However, text exercises were considered useful for recapitulation and therefore both formats were needed.

Eight participants (53%) performed exercises in the order recommended by Oiva. Guidance was perceived to facilitate use, as reflected in a comment: “It does not limit but provides the direction where to go, that works!” If the participants felt that the recommended exercise was not suitable for a specific situation, they skipped it at that moment. The recommended order was not taken by 47% (7/15) of participants, who chose exercises based on interesting topics, names of the exercises, or their own feelings. They did not feel that the guidance limited their freedom of choice. The app “told in a plain enough way, not with exclamation mark, where one is going and what is being suggested next”, as one participant articulated.

Although the participants liked the current guidance in Oiva, they wished for even more guidance. Six participants (40%) would have liked a scheduled program and 67% (10/15) would have liked to receive reminders to use Oiva. They felt that these features would bring order and motivation. However, they stated that scheduling should not be too restrictive. Skipping and choosing different exercises should still be possible.

Skepticism toward gamification was expressed by 60% (9/15) of participants. They thought that collecting points, rewards, and achievements would not sit well with a mental wellness app or fit the philosophy of mindfulness. They felt that the motivation to use Oiva rises from its content and wellness effects and that the real achievements and rewards come from learning and self-improvement. Instead, they wanted to see their progress in the skills they had learned and the positive changes they had accomplished.

Nine participants (60%) made entries to Oiva’s diary. The interviews revealed that the entries mainly dealt with thoughts, feelings, and insights raised by the exercises, and answers to the questions in the reflection screen. According to the participants, one of the main benefits of the diary was that it enabled the follow-up of thoughts when the same exercise was repeated. Two participants used a paper diary, because they wanted to be able to write and draw freely. Three participants wished to have more structured and guided questionnaires with pre-defined response options, as they found it difficult to know what to write. They also felt that a structured diary would enable easier follow-up and comparison of entries. In general, the touch screen text input was regarded as cumbersome, which may have caused some of the problems encountered with the diary.

At the time of the study, Oiva did not offer anything extra when the user had completed all the exercises, which 80% (12/15) of the participants saw as an inadequate end for the program. Nine participants (60%) mentioned that they would have wanted a follow-up program to remind them to continue to do the exercises, preferably focusing on the exercises that were the most beneficial for them or where there still was room for improvement. The follow-up program could also provide new content and exercises. The participants felt that the usage of Oiva should not end after completing the exercises once, but that the exercises should be repeated whenever needed. The skills learned should be integrated to one’s life and Oiva could help by guiding in that.

## Discussion

### Principal Findings

The 4 main goals of this study were: (1) to find out how the participants used the app, (2) to study whether using Oiva improved the participants’ mental wellness, (3) to explore how Oiva and its functions were experienced, and (4) to derive preliminary design guidelines for mobile stress management apps.

Oiva was used actively by the participants, on average, every third day. The sessions were relatively long for mobile use, on average 12 minutes. This result is similar to the one reported by Doherty et al [32], albeit their intervention was Web-based. Although daily use of Oiva was recommended to the participants, finding personally appropriate ways of use was also encouraged. Longer sessions may be useful for learning the skills at the beginning, whereas later on, short sessions may be enough to maintain them. This indicates that different phases of use should be identified and supported.

Statistically significant improvements in stress and life satisfaction were achieved during the study. The positive mean ratings on the subjective scales of improvement or maintenance of wellness, learning new things, and gaining new insights suggest that the participants explicitly attributed these positive effects to Oiva, although other factors (eg, changes in common stressors for the university staff) cannot be excluded in this study. Furthermore, as the study sample was small, these results should be considered preliminary and indicative.

The interview data implied that the participants had taken the first steps towards learning the skills related to ACT. Most participants reported increased mindfulness, but also learning other skills, such as skills related to acceptance, cognitive defusion, values, and committed actions. However, we did not observe significant changes in psychological flexibility, which would have been in line with such changes. One of the reasons may be that most of the sample already had some prior knowledge of ACT. Moreover, psychological flexibility has been described as a fundamental basic aspect of health [41], and changes in such basic psychological processes may take a longer period of time to manifest. However, there is evidence that gaining changes in psychological flexibility within such a short time period may be possible by concentrating on specific processes, such as values and committed actions, as in the work by Ly et al [24]. However, in contrast to our results, they did not find any changes in life satisfaction, which may be due to their narrower coverage of ACT processes. In our study, most of the participants started with mindfulness exercises and did not have enough time to properly go through exercises related to acceptance and values, which could have a more direct impact on psychological flexibility. The comparison of the outcomes of these two studies poses an interesting question: does focusing on different processes of ACT influence different aspects of psychological wellness?

### Design Implications

It is assumed that mobile devices are well-suited for wellness apps because they enable interventions in mobile contexts. In our study, we found that most of the use occurred, not in mobile situations, but in situations that were peaceful and provided an opportunity for proper concentration. The short breathing and relaxation exercises formed an exception—they were found easy to integrate into everyday life and in some cases even mobile situations. However, the strengths of the mobile platform became evident because the mobile phone was easy to carry along anywhere enabling more freedom in choosing the locations of use. Also, being fast to open for a quick session was an important benefit.

Participants appreciated guidance in both navigation and performing the exercises. They liked to hand over the responsibility of deciding the order of exercises to the app. Thus, guidance may have helped in creating a sense of therapeutic alliance between Oiva and the participants [31].

Based on our findings, we propose the following set of preliminary design implications for mobile mental wellness training apps.

#### Provide Exercises for Everyday Life

Most, if not all, participants understood that the purpose of Oiva was to learn skills and techniques that improve wellness, and to integrate these skills to everyday life as a process of mental self-development. The main benefit for most participants was the possibility to do exercises to calm down quickly. The short breathing and relaxation exercises were used to take a break, a “moment for me”. Such exercises were well-suited to the relatively short one-month study period, since they were quick to absorb, easy to integrate into one’s daily habits and everyday life, and did not require much thought or preparation.

#### Find Proper Place and Time for Challenging Content

The participants perceived some of Oiva’s exercises as more challenging, especially the exercises that were longer or related to more challenging skills (eg, values or acceptance). They required more concentration and effort, and therefore had a high threshold to get started with. However, the challenging exercises would probably have stronger effects on psychological flexibility and thus it is essential to lower the threshold for the users to engage in them. One way to approach this could be to utilize context-awareness on mobile platforms to identify appropriate moments to engage the user [42]. In the future, the context-aware system could be able to recognize a suitable time and a peaceful place for performing the exercises requiring more concentration, and prompt the user at that moment. Another less technical solution would be to provide an opportunity for the user to filter and search exercises for specific contexts and needs.

#### Focus on Self-Improvement and Learning Instead of External Rewards

Interestingly, the majority of the participants did not wish for extra rewards or game-like elements in Oiva. An achievement-oriented approach is often utilized in the apps for physical activity [30], and it is easy to assume that users would enjoy a gamified approach also in mental wellness apps. However, our participants thought otherwise. Playful interface or hunting for rewards was not seen to fit the philosophy of mindfulness, concentration, and calming down. The participants felt they were rewarded and intrinsically motivated by learning new skills and seeing changes in their lives. This is in line with the notion that it is more important to focus on meaningful experiences than rewards [43]. As argued by Doherty et al [32], the designers should emphasize the engagement with the treatment, rather than with the technology. However, at the same time we note that our participants were working age adults in a university setting. Rewards and a gamified approach might suit other types of user profiles better.

#### Guide Gently but Do Not Restrict Choice

In general, the preference for guidance as “giving direction but not limiting” was expressed frequently. Oiva’s way of gently recommending an exercise while still leaving the user the freedom of choice was appreciated. The participants also liked having audio narration to guide them through exercises. In self-help apps, active guidance is a way to foster a sense of alliance [31]. Even more guidance was wished for in the form of a scheduled program in the calendar and reminders for specific exercises. Furthermore, some implied that a follow-up program would be useful to integrate the skills learned in the intervention as a part of life. Based on these findings, it seems that the participants wanted to have a feeling of being guided through the program, but they did not want to follow too strict tunnels without an option to skip exercises or select more suitable ones. Therefore, the persuasive design principle of tunneling (narrowing down the choices available for the user) [26,27] should not be used excessively.

#### Provide an Easy and Flexible Tool for Self-Reflection

Many participants used the diary as a self-reflection tool, as they wrote down feelings and thoughts related to exercises, as well as responded to the questions that were asked at the end of the exercises. The participants emphasized the importance of raising emotional awareness through self-reflection [31]. Some of them desired a diary with structured questionnaires and pre-defined answers, revealing the need for a wider range of interactivity [32]. Not surprisingly, a mobile device was not considered ideal for free text input. Considering the successful use of self-tracking and mood monitoring in previous studies [19,20], a structured manner of recording feelings and emotions as well as ability to view historical data could be useful.

### Limitations

This study was an uncontrolled field trial involving a small number of volunteer participants, which must be taken into account in interpreting the results. Many of the participants were already somewhat familiar with ACT-based methods, which may have facilitated the adoption of the app and learning the skills. Due to the lack of a control group, common confounding factors affecting the wellness of university staff at the time of the study cannot be assessed. Also the usage patterns observed in the study may not fully reflect realistic usage patterns, as the participants were not able to use Oiva in their own mobile phones.

The study participants were not the direct target group of the app. For ethical reasons, we did not want to present a prototype app to people suffering from severe stress, and thus chose a healthy group of participants for this feasibility study. This choice limits the generalizability of the results.

### Conclusions and Future Work

This article presented Oiva, a mobile stress management app based on acceptance and commitment therapy methods. We studied the usage, impact, and user experiences of Oiva in a one-month field study with 15 participants. The active usage, observed positive effects on wellness, and the generally positive user experiences of Oiva suggest that it is possible to develop engaging mobile apps that are experienced as beneficial for personal mental wellness. Our present results establish Oiva as a good starting point for continuing research on mobile support for mental wellness. We believe that the insights gained from our in-depth interviews with the participants may help future researchers to create effective and engaging mental wellness apps.

Oiva is currently being studied in a randomized controlled trial with working-age subjects suffering from stress and features of metabolic syndrome. In the study, Oiva is compared to an ACT-based face-to-face intervention with similar content, delivered as 6 group meetings during an 8-week period, and a control group. Each study condition will involve about 80 subjects. The impact of interventions will be measured by psychological, physical, and anthropometric questionnaires and measurements at baseline, after the intervention, and at 6 months after the intervention.

In future versions of Oiva, we aim to enable easier tailoring of programs to different target groups as well as individual needs and explore the possibilities of context-sensitivity in supporting better integration into the daily life. We will also study the opportunities of adding social features to the app [31,32]. However, our aim is to continue to develop and study Oiva in an iterative way, making sure that the existing features are successful before adding new ones.

## Abbreviations

AAQ-II: acceptance and action questionnaire
ACT: acceptance and commitment therapy
CBT: cognitive behavioral therapy
SEM: standard error of mean
SWLS: satisfaction with life scale
